# Efficacy of Transdermal Oxybutynin in the Treatment of Overactive Bladder Syndrome: Does It Make Sense Using It in 2017?

**DOI:** 10.1155/2018/6782736

**Published:** 2018-07-29

**Authors:** Raúl Vozmediano-Chicharro, Blanca Madurga, Pedro Blasco

**Affiliations:** ^1^Department of Urology, Hospital Regional de Málaga, Hospital Civil, University of Malaga, Plz Hospital Civil s/n, 29009 Malaga, Spain; ^2^Department of Urology, Hospital Universitario Puerta del Mar, Av. Ana de Viya 21, 11009 Cadiz, Spain; ^3^Department of Urology, Hospital Universitario de Valme, Ctra. de Cádiz Km. 548.9, 41014 Seville, Spain

## Abstract

**Objectives:**

Evaluation of changes in symptoms among patients with overactive bladder syndrome treated with transdermal oxybutynin and tolerability after 12 months of follow-up.

**Methods:**

This was a multicenter, retrospective, single-cohort, observational study. Changes in symptoms were evaluated primarily with a 3-day voiding diary. Results were compared to baseline. Subgroup analyses were performed in patients previously treated for OAB or not and aged < 65 years versus ≥65 years.

**Results:**

Clinical records of 105 patients were examined; 92.4% were women. At 12 months, 58 patients continued to receive transdermal oxybutynin. Changes in symptoms according to the voiding diary were evaluated in 47 patients. Significant improvements from baseline were observed in urinary frequency (−2.6 voids/24 hours (95% CI: −3.5; −1.8), *p* < 0.001); daily number of urgent episodes (−4.7 episodes/day (95% CI: −6.1; −3.6), *p* < 0.001); and urge incontinence (−1.9 episodes/day (95% CI: −2.9; −1.3), *p* < 0.001). No statistically significant differences were found in subgroup analyses. In total, 38.1% of patients had adverse events, primarily in the application site (27.6%). No severe systemic adverse events occurred. Only 6 patients (5.7%) reported dry mouth.

**Conclusions:**

Improved symptoms and good tolerability observed after 1 year of treatment with transdermal oxybutynin shows that it currently has a place in the treatment of OAB patients.

## 1. Introduction

Overactive bladder syndrome (OAB) has been clinically defined as urinary urgency, with or without incontinence, generally accompanied by an increase in urinary frequency and nocturia, after any local disease or metabolic disorder that would explain these symptoms has been ruled out [1]. According to the EPIC study in 2009, the prevalence of overactive bladder and/or urinary incontinence in Spain reached 54% in subjects over the age of 65 [2]. The number of individuals with OAB is expected to increase in successive years as the population ages.

Despite the benefits of antimuscarinics in OAB treatment [3, 4], oral administration causes well-known side effects, in particular dry mouth, often leading patients to discontinue treatment and tolerate their incontinence [5]. Administration of transdermal oxybutynin (OXY-TDS) was approved by the Spanish Medicine Agency in 2004. Transdermal delivery has been shown to significantly reduce side effects by decreasing the active metabolite of oxybutynin (N-DEO) involved in their onset, thus possibly improving treatment adherence [6]. The efficacy and safety of OXY-TDS in the treatment of OAB have been established in several clinical trials [7–9].

Although randomized clinical trials are still the gold standard, generalization of results from these studies may be limited by widely varying mid- and long-term responses in real-world situations, lack of treatment adherence, or the use of drugs in patients with diverse morbidities. Therefore, the aim of this study was to ascertain if the improvement in symptoms and good tolerability shown in clinical trials are reproduced under clinical practice conditions.

## 2. Methods

The Oxybutynin-transdermal in Standard ClinicAl pRactice study (OSCAR study) was a retrospective cohort study in clinical practice conditions. Clinical records of patients with a diagnosis of OAB who received OXY-TDS in 3 centers in the same Spanish health-care region were reviewed. Data were collected at baseline, before starting treatment with OXY-TDS, and at 6 and 12 months of treatment. Adult patients of both sexes with a diagnosis of OAB according to their clinical records and a 3-day voiding diary (3 DVD) who had started treatment with OXY-TDS at least 12 months before inclusion in the study were enrolled. The study was conducted in accordance with the ethical requirements of the Declaration of Helsinki and Good Clinical Practice guidelines and approved by the Provincial Research Ethics Committee of Malaga.

The primary objective was to evaluate changes in symptoms in OAB patients receiving OXY-TDS after 12 months of treatment. Tolerability to OXY-TDS was also evaluated.

Changes in symptoms were evaluated using the 3 DVD as the primary tool. Parameters included in the 3 DVD are shown in Table 1. The urgency visual analogue scale (VAS) was a 10-point Likert-type scale, the OAB-V8 scale for detection of symptomatic OAB [10], and the Spanish validation of the International Consultation on Incontinence Questionnaire-Short Form (ICIQ-SF) [11] were also used. Tolerability was evaluated from adverse events recorded, percentage of treatment interruptions, and reasons for discontinuation.

Subgroup analyses were also performed to compare changes in 3 DVD parameters among patients treated (nonnaive) versus not previously treated (naive) with OXY-TDS for OAB and patients aged less than 65 years versus 65 years or more.

Sample size was based on an alpha risk of 5% and a statistical power of 80% for comparing 24-hour urinary frequency pre- and posttreatment with OXY-TDS. A minimum of 118 subjects were estimated to be necessary for detecting statistically significant differences around 10% between baseline and end of the treatment. A baseline voiding frequency of 11 was assumed, along with a standard deviation of 4.

The primary statistical analysis was based on a comparison of clinical outcomes between the OXY-TDS pre- and posttreatment periods (baseline versus 12 months) in patients who completed the study correctly (per protocol analysis). The baseline status was also compared with status after 6 months of OXY-TDS treatment to evaluate an interim response between the different study parameters.

Data were described by counts and percentages, mean and standard deviation, or median and minimum and maximum, as appropriate. Pre- to posttreatment results were compared by paired *t*-test, Wilcoxon's signed rank test, McNemar's test, or the McNemar-Bowker marginal homogeneity chi-squared test, as appropriate.

Statistical tests were performed using SPSS version 20.0 (IBM SPSS, Inc., Chicago, IL, USA) and Stats Direct software (V.2.s8).

## 3. Results

**Figure 1:**
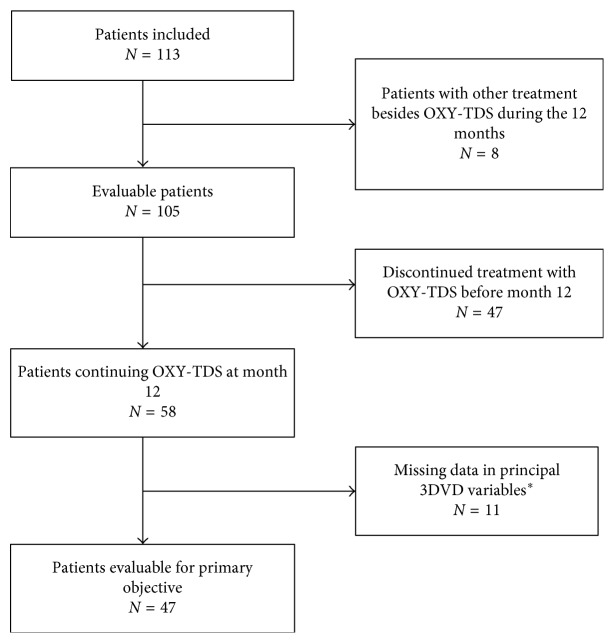
Study flow chart. Patients evaluated for study objectives according to data collected in clinical records. ^*∗*^Frequency, urgency, and urge incontinence. OXY-TDS: transdermal oxybutynin. 3 DVD: 3-day voiding diary.

Clinical records of 105 patients who met the study selection criteria were evaluated.  Figure 1 shows the study flow chart. All patients included in the study were Caucasian, 92.4% were women (7.62% men). Age was 59.4 (11.8) (mean and SD) years, and body mass index was 26.8 (4.4). Time since onset of OAB before treatment with OXY-TDS was 4.10 (4.9) years. The most common clinical antecedents were depression in 18 patients (17.1%) and surgical interventions for urinary incontinence in 19 patients (18.1%). Overall, of the patients for whom data was available, 66 patients (66.7%) had received previous treatment for OAB before starting OXY-TDS. Anticholinergics were the most common, followed by beta-3 agonists, and in both cases, the most common reason for discontinuation was lack of efficacy, although adverse events were also a very common cause of discontinuation of anticholinergics. All previous treatments were discontinued before the start of OXY-TDS except for 1 patient who was taking a beta-3 agonist.

After 6 months of treatment, 63 patients (60.0%) continued to receive OXY-TDS, 45 of whom provided 3 DVD data. Reductions were observed in the number of daytime, night-time, and 24-hour urinary episodes, number of episodes of urgency and urgency grade, and the number of changes of underwear or protective pads. All these changes were statistically significant.

Twelve months after initiating treatment, 58 patients (55.2%) continued to receive OXY-TDS. Complete 3 DVD data were available at 12 months from 47 of 58 patients (81.0%). Statistically significant improvements from baseline were observed in the number of voids, both during the day and at night, in the number of daily urinary urgency and urgent urinary episodes and in urinary urgency grade. Statistically significant changes were also observed in the number of times a patient needed a change of underwear or absorbent pads (Table 1). A total of 33 patients (70.2%) presented more than 4 episodes of urgency/day at baseline, and this proportion fell to 2 of 47 patients or 4.2% after 12 months of treatment with OXY-TDS (*p* < 0.001).

Although all patients completed the urgency VAS (*N*=58) at 12 months, the evaluation of the 3 DVD completed by patients (*N*=47) showed a statistically significant improvement from baseline (−4.50 points (95% CI: −5.50; −4.00), *p* < 0.001). The difference in the mean ICIQ-SF scale at baseline and after 12 months of treatment was −8 points (95% CI: −10.0; −6.5, *p*=0.000) (Table 2), while on OAB-V8, the difference in score was −13.5 (95% CI: −16.0; −11.5, *p* < 0.001).

No statistically significant differences were observed in any of the 3 DVD variables at 12 months of treatment between patients who were not previously treated for OAB (naive) and patients who had been treated (nonnaive). Nor were statistically significant differences observed between patients who were younger than 65 years and those who were 65 and older (Tables 3 and 4).

Twelve months after starting treatment, 47 patients (44.8%) had discontinued treatment. Reasons for discontinuation were adverse events in 18 patients (38.3%), lack of response in 17 (36.2%), and lack of treatment compliance in 4 (8.5%); another reason or no reason was given for 8 (17.0%) patients.

Regarding tolerability, 40 patients (38.1%) had some kind of adverse event at 12 months, mainly at the site of application of the patch (29 patients, 27.6%), 16 (15.2%) in the form of pruritus, 4 (3.8%) with erythema, and 13 (12.4%) with irritated skin. Two of the local adverse events (skin irritation) were clinically significant and consequently considered severe. Dry mouth and constipation were recorded in only 6 (5.7%) and 1 (0.95%) of the patients, respectively.

## 4. Discussion

In this study, we examined retrospective data from the real-world management of patients with OAB treated with OXY-TDS over a 12-month period. Similarly to other studies reported in the literature [8, 9, 12, 13], we found that patients receiving OXY-TDS showed a reduction in OAB symptoms, with statistically significant changes in symptoms according to the 3 DVD, and in the grade of urgency perceived by patients, as recorded on the specific urgency VAS. The combined analysis performed by Dmochowski et al., in particular, found that OXY-TDS was more effective than placebo in the reduction of daily incontinence episodes (−3.0 versus −2.0, *p*=0.0004) and daily urinary frequency (−2.0 versus −1.0, *p*=0.0023) [12]. Moreover, OXY-TDS showed reductions similar to other antimuscarinic drugs. Findings from a review of randomized controlled trials with antimuscarinic drugs for OAB conducted by Novara et al. included reductions in urinary frequency (between −0.7 and −3.69), urgency episodes (between −1.0 and −3.4), or urgency urinary incontinence (between −0.3 and −2.28) [3]. It is also important to note that in our study, almost all incontinent patients (4 episodes of urgency/day) before treatment became continent after being treated with OXY-TDS.

In this study, we observed an improvement in symptoms due to OXY-TDS in the first 6 months, which was maintained until the end of the 12-month observational period. This is in line with results from clinical trials, which reported a response in the early weeks of treatment that was maintained during the following period [6].

Results suggest no differences in response to treatment between patients who were previously treated or treatment-naive. Similarly, no differences were found between patients younger and older than 65 years of age, but further studies are required to confirm these findings. In this study, approximately half of the patients received previous treatment and continued to present symptoms, and this observation is similar to those of the MATRIX study, despite the current availability of drugs such as solifenacin and mirabegron.

The most troublesome symptoms of OAB are urgency and urge incontinence [14], which may affect work productivity, impact negatively on quality of life, and lead to depression [15]. Similarly, alleviating OAB symptoms can have a significantly positive effect on health-related quality of life [16]. In this study, the overall improvement in 3 DVD and urgency VAS scores, along with improvements in all domains evaluated in both the ICIQ-SF questionnaire and OAB-V8 scale, affirm that our patients achieved an overall improvement in quality of life. Seventeen percent of our patients had depression, so long-term follow-up studies would be of interest to study associations between improvements in OAB symptoms and improvements in depressive syndromes.

VAS is a widely accepted research tool for measuring the impact of a disease and the effects of medical interventions on quality of life and has also been useful in urinary incontinence studies. It has been used for both evaluating the effects of urinary incontinence treatments and estimating the subjective perception of the patient with regard to their urinary incontinence [15, 17, 18]. In this study, the urgency VAS tool demonstrated its ability to measure treatment response. More completed urgency VAS scales (*N=*58) than completed 3 DVDs3D (*N=*47) were available in the patients' clinical records, and this might be interpreted as greater acceptance of the urgency VAS tool by patients, due to its simplicity as a measure of perceived grade of incontinence. Although 3 DVD is the reference instrument in clinical trials, in clinical practice, it is very difficult for patients to use. However, the urgency VAS may be a transparent, simple-to-use instrument that is easy for the clinician to interpret.

In our study, dry mouth occurred at a rate of 5.7%, less prevalent than expected in the Summary of Product Characteristics [19] (8.6%) and with other antimuscarinic drugs. In the review by Novara et al., the adverse event of dry mouth was between 13% and 86% depending on the study and antimuscarinic drug [3].

The MATRIX study, a 6-month phase IV trial carried out in the US in patients from urology, primary care, gynecology, and geriatrics departments, evaluated changes in health-related quality of life and work productivity in patients with OAB treated with OXY-TDS and found significant improvements in study parameters, along with good tolerability [20]. Two reviews of oxybutynin concluded that the transdermal formulation is generally well tolerated by OAB patients, with a low rate of anticholinergic-related adverse events, most of which were local, such as skin irritation at the site of application of the patch [6, 21]. The transdermal form of oxybutynin may offer a better tolerated therapeutic alternative to patients with OAB and urge incontinence in the management of their symptoms and thus may improve treatment adherence by patients [5, 20, 22].

In line with the MATRIX study [20], in which dermatitis, in the form of irritation, redness, and pruritus at the site of application of the patch, occurred in 14.0% of patients, adverse events related with site of application were also the most common in our study. Neither of the local adverse events (skin irritation) considered severe because of their clinical significance required hospital admission nor they did produce disability; in 1 case, the patient had a history of atopic dermatitis that she had not reported previously.

Treatment adherence with OXY-TDS was high in preapproval clinical trials, in which 86–87.1% of participating patients completed the entire study treatment period [8, 9]. In our study, conducted in a standard clinical practice setting, 55.2% of the patients were continuing treatment at 12 months. Adverse events and failure to respond as expected were the most common reasons for discontinuation, in line with published reviews [23]. It is important to highlight that the persistence rate achieved in our study is high, and at least as high as that with other treatments options (23.7–66%), possibly associated with differences in efficacy and tolerability [24–27].

In general, however, adverse events were not serious, and treatment discontinuation could suggest that for many patients, the treatment benefits do not outweigh the drawbacks. Further studies are needed to gain more insight into how patients weigh up the difference between expected response and adverse events and what leads them to decide to maintain or discontinue a certain treatment.

To our knowledge, the OSCAR study is the first real-world study carried out in Europe recruiting patients exclusively from urology departments. In the MATRIX study, a 6-month phase IV trial carried out in the US including patients from different departments, the primary endpoint was changes in quality of life, but the 3 DVD was not used for evaluating effectiveness, even 3D as a secondary endpoint. The OSCAR study evaluated changes in signs and symptoms at 12 months as a primary endpoint, using a patient urinary diary and the OAB-V8 and ICI-Q questionnaires. In addition, cognitive function, adherence, and satisfaction were evaluated as secondary endpoints.

The limitations of this study are those typical of retrospective cohort studies and include among others bias in patient selection and missing data that might affect the final results. Nevertheless, in order to reduce possible bias, strict criteria were applied to the selection of patients, and data were evaluated for each variable according to the number of patients from whom those data were available. It is probable that patients who continue treatment have better results in terms of improvement of symptoms; however, this study did not evaluate symptoms in patients who discontinued. Another possible limitation is that the 3 participating sites were not randomly selected and results may not reflect the reality observed in other regions of Spain. However, that may be an advantage since all 3 centers belonged to the same health-care region that uses a standardized record system.

In conclusion, in view of the results of OSCAR study, it seems that there is currently an important place for OXY-TDS in the treatment of OAB, although further real-life studies should be carried out to extend knowledge of the use and effects of the drug in real practice.

## Figures and Tables

**Table 1:** Changes from pre- to posttreatment in 3 DVD parameters after 12 months of OXY-TDS treatment.

3 DVD parameters	Pretreatment		Posttreatment		Change (95% CI)	*p* value
	*N*	*M* (SD)	*N*	*M* (SD)		
DUF (episodes/day)^*∗*^	47	9.1 (3.1)	46	7.0 (1.6)	−1.7 (−2.55;−1.15)	<0.001
NUF (episodes/night)^*∗*^	47	1.6 (1.4)	44	0.8 (0.8)	−0.9 (−1.12;−0.45)	<0.001
UF/24 h (episodes/day)^*∗*^	47	10.7 (3.9)	47	7.7 (1.8)	−2.6 (−3.50;−1.80)	<0.001
Maximum UV (ml)^*∗*^	38	250.7 (107.8)	39	346.0 (278.5)	63.3 (19.95; 105.85)	0.006
Nocturnal UV (ml)	38	146.6 (134.1)	39	134.2 (147.7)	−16.2 (−81.65; 48.35)	0.651
Mean UV (ml)	5	134.8 (83.4)	5	202.6 (47.9)	0.8 (−22.30; 20.70)	0.976
Urgency (episodes/day)	47	7.0 (4.7)	47	1.5 (1.5)	−4.7 (−6.05;−3.55)	<0.001
Urgency grade^*∗*^	47	3.7 (0.5)	44	2.3 (1.3)	−1.4 (−1.85; −0.85)	<0.001
UUI (episodes/day)^*∗*^	46	2.7 (2.7)	43	0.5 (0.9)	−1.9 (−2.85; −1.30)	<0.001
SUI (episodes/day)^*∗*^	47	0.7 (1.2)	47	0.2 (0.5)	0.0 (−0.85; 0.00)	0.007
Pad/underwear changes (number/day)^*∗*^	40	3.4 (4.1)	45	0.7 (1.1)	−2.2 (−2.80; −1.50)	<0.001
Liquid intake/24 h (ml)	38	1435.1 (406.1)	38	1453.5 (518.9)	15.8 (−134.15; 179.95)	0.698
Urine output/24 h (ml)	38	1385.9 (381.2)	39	1504.4 (381.1)	115.9 (0.00; 223.35)	0.054^*∗∗*^
Nocturnal urine output (ml)	37	200.8 (161.5)	38	174.7 (202.4)	−30.9 (−107.50; 34.20)	0.51

3 DVD: 3-day voiding diary; DUF: daytime urinary frequency; NUF: night-time urinary frequency; UF/24 h: 24-hour urinary frequency (total number of episodes/24 hours); SUI: stress urinary frequency; UV: urinary volume; UUI: urge urinary frequency. *p* values: Student's *t*-test or ^*∗*^Wilcoxon signed rank test. Change: mean difference or ^*∗*^Hodges-Lehman estimator of location shift. ^*∗∗*^This variable was not reported in the 6-month visit in any of the evaluable cases. For this reason, no statistical comparisons were performed.

**Table 2:** ICIQ-SF questionnaire. Changes from baseline after 12 months of treatment with OXY-TDS.

ICIQ-SF questionnaire	Pretreatment patients *N*=47	Posttreatment patients *N*=47	*p* value^*∗∗*^
	*n*	*n*	
No urine leaks	2 (4.3%)	18 (38.3%)	<0.001
Leaks before you can get to the toilet	41 (87.2%)	22 (46.81%)	<0.001
Leaks when you cough or sneeze	18 (38.30%)	7 (14.9%)	0.013
Leaks when you are asleep	11 (23.4%)	3 (6.4%)	0.008
Leaks when you are physically active/exercising	16 (34.04%)	7 (14.9%)	0.035
Leaks when you have finished urinating	9 (19.15%)	2 (4.3%)	0.039
Leaks for no obvious reason	15 (31.91%)	2 (4.3%)	<0.001
Leaks all the time	2 (4.25%)	0 (0.0%)	NA

ICIQ-SF item 1 and 2 = 0	2	19	<0.001
ICIQ-SF item 1 or 2 > 0	45	27	

ICIQ-SF = 0	2	12	0.002
ICIQ-SF > 0	45	34	
	*M (SD)*	*M (SD)*	*Change (95% CI)*
Total score	13.40 (4.5)	5.13 (4.5)	−8 (−10.0; −6.5) *p* value 0.000^*∗*^

Statistical tests: Student's *t*-test (variables with normal distribution) or Wilcoxon signed rank test (^*∗*^variables with nonnormal distribution); ^*∗∗*^McNemar.

**Table 3:** Changes 3D from baseline in the 3 DVD tool at 12 months of treatment with OXY-TDS (naive/nonnaive patients).

3 DVD	Naive (*n*=15) mean (SD)	Nonnaive (*n*=32) mean (SD)	Diff (95% CI)	*p* value
DUF (episodes/day)^*∗*^	7.07 (1.8)	6.9 (1.5)	0.4 (−0.7; 1.3)	0.341
NUF (episodes/night)^*∗*^	0.87 (0.7)	0.7 (0.8)	0.0 (−3.0; 0.7)	0.411
UF/24 hours (episodes/day)^*∗*^	8.0 (2.1)	7.6 (1.7)	6.3 (−0.4; 1.7)	0.298
Maximum UV (ml)^*∗*^	444.6 (486.1)	302.1 (83.1)	24.1 (−41.7; 100.0)	0.461
Nocturnal UV (ml)	112.6 (84.9)	143.83 (168.9)	−31.2 (−135.9; 73.5)	0.550
Mean UV (ml)	205.5 (54.9)	191.0 (NA)	14.5 (−180.6; 209.6)	0.828
Urgency (episodes/day)	1.5 (2.1)	1.5 (1.2)	0.5 (−0.9; 1.0)	0.892
Urgency grade^*∗*^	1.8 (1.3)	2.5 (1.3)	−0.7 (−1.3; 0.0)	0.071
UUI (episodes/day)^*∗*^	0.2 (0.5)	0.6 (1.0)	0.0 (−0.3; 0.0)	0.328
SUI (episodes/day)^*∗*^	0.0 (0.0)	0.2 (0.6)	0.0 (0.0; 0.0)	0.110
Pad/underwear changes (number/day)^*∗*^	0.6 (1.0)	0.8 (1.1)	0.0 (−0.6; 0.0)	0.044
Liquid intake/24 h (ml)	1484.73 (613.5)	1439.0 (481.9)	45.7 (−326.4; 417.1)	0.805
Urine output/24 h (ml)	1609.17 (446.6)	1457.89 (347.2)	151.3 (−115.5; 418.0)	0.258
Nocturnal urine output (ml)	167.77 (138.3)	185.26 (228.2)	−17.5 (−162.6; 127.6)	0.808

3 DVD: 3-day voiding diary; DUF: daytime urinary frequency; NUF: night-time urinary frequency; SD: standard deviation; SUI: stress urinary incontinence; UF/24 h: 24-hour urinary frequency (total number of episodes/24 hours); UUI: urge urinary incontinence. *p* values: Student's *t*-test or ^*∗*^Wilcoxon signed rank test. Change: mean difference, or ^*∗*^Hodges-Lehman estimator of location shift.

**Table 4:** Changes 3D from baseline in the 3 DVD at 12 months of treatment with OXY-TDS (patients < 65 years and ≥65 years).

3 DVD	<65 years (*n*=33) mean (SD)	≥65 years (*n*=14) mean (SD)	Diff (95% CI)	*p* value
DUF (episodes/day)^*∗*^	6.83 (1.5)	7.2 (1.9)	−0.3 (−1.3; 0.7)	0.615
NUF (episodes/night)^*∗*^	0.6 (0.7)	1.1 (0.9)	−0.3 (−1.3; 0.0)	0.053
UF/24 h (episodes/day)^*∗*^	7.4 (1.6)	8.4 (2.2)	−0.7 (−2.0; 0.3)	0.129
Maximum UV (ml)^*∗*^	373.5 (328.4)	284.0 (203.7)	40.0 (−26.7; 106.7)	0.221
Nocturnal UV (ml)	107.4 (109.2)	194.6 (194.4)	−87.2 (−188.3; 14.0)	0.089
Mean UV (ml)	205.5 (54.9)	191 (n/a)	—	—
Urgency (episodes/day)	1.5 (1.6)	1.5 (1.2)	−0.1 (−1.0; 0.9)	0.876
Urgency grade^*∗*^	2.3 (1.3)	2.3 (1.4)	0.0 (−0.7; 0.7)	0.805
UUI (episodes/day)^*∗*^	0.3 (0.5)	0.87 (1.4)	0.0 (−0.7; 0.0)	0.232
SUI (episodes/day)^*∗*^	0.1 (0.2)	0.3 (0.9)	0.0 (0.0; 0.0)	0.514
Pad/underwear changes (number/day)^*∗*^	0.4 (0.6)	1.5 (1.5)	−0.7 (−2.0; 0.0)	0.024
Liquid intake/24 h (ml)	1431.2 (522.3)	1508.2 (531.3)	−77.0 (−457.8; 303.7)	0.684
Urine output/24 h (ml)	1503.4 (377.6)	1506.8 (405.3)	−3.4 (−274.9; 268.1)	0.980
Nocturnal urine output (ml)	127.0 (132.7)	309.2 (282.4)	−182.3 (−317.7; 46.8)	0.063

3 DVD: 3-day voiding diary; DUF: daytime urinary frequency; NUF: night-time urinary frequency; SD: standard deviation; SUI: stress urinary incontinence; UF/24 h: 24-hour urinary frequency (total number of episodes/24 hours); UV: urinary volume; UUI: urge urinary incontinence. *p* values: Student's *t*-test or ^*∗*^Wilcoxon signed rank test. Change: mean difference or ^*∗*^Hodges-Lehman estimator of location shift.

## Data Availability

Access to the clinical data used to support the findings of this study, being derived from hospital medical records, is restricted by the Provincial Research Ethics Committee of Malaga in order to protect patient privacy.
